# Lack of Association Between Genetic Variants at *ACE2* and *TMPRSS2* Genes Involved in SARS-CoV-2 Infection and Human Quantitative Phenotypes

**DOI:** 10.3389/fgene.2020.00613

**Published:** 2020-06-08

**Authors:** Esteban A. Lopera Maya, Adriaan van der Graaf, Pauline Lanting, Marije van der Geest, Jingyuan Fu, Morris Swertz, Lude Franke, Cisca Wijmenga, Patrick Deelen, Alexandra Zhernakova, Serena Sanna, Lifelines Cohort Study

**Affiliations:** ^1^Department of Genetics, University of Groningen, University Medical Center Groningen, Groningen, Netherlands; ^2^Department of Pediatrics, University of Groningen, University Medical Center Groningen, Groningen, Netherlands; ^3^Department of Genetics, University Medical Centre Utrecht, Utrecht, Netherlands; ^4^Institute for Genetics and Biomedical Research (IRGB), Consiglio Nazionale delle Ricerche (CNR), Monserrato, Italy

**Keywords:** PheWAS, *ACE2*, *TMPRSS2*, NSAIDs (non-steroidal anti-inflammatory drugs), ARBs (angiotensin II receptor blockers), COVID-19, SARS-CoV-2

## Abstract

Coronavirus disease 2019 (COVID-19) shows a wide variation in expression and severity of symptoms, from very mild or no symptoms, to flu-like symptoms, and in more severe cases, to pneumonia, acute respiratory distress syndrome, and even death. Large differences in outcome have also been observed between males and females. The causes for this variability are likely to be multifactorial, and to include genetics. The SARS-CoV-2 virus responsible for the infection depends on two human genes: the human receptor angiotensin converting enzyme 2 (*ACE2*) for cell invasion, and the serine protease *TMPRSS2* for S protein priming. Genetic variation in these two genes may thus modulate an individual's genetic predisposition to infection and virus clearance. While genetic data on COVID-19 patients is being gathered, we carried out a phenome-wide association scan (PheWAS) to investigate the role of these genes in other human phenotypes in the general population. We examined 178 quantitative phenotypes including cytokines and cardio-metabolic biomarkers, as well as usage of 58 medications in 36,339 volunteers from the Lifelines population cohort, in relation to 1,273 genetic variants located in or near *ACE2* and *TMPRSS2*. While none reached our threshold for significance, we observed several interesting suggestive associations. For example, single nucleotide polymorphisms (SNPs) near the *TMPRSS2* genes were associated with thrombocytes count (*p* = 1.8 × 10^−5^). SNPs within the *ACE2* gene were associated with (1) the use of angiotensin II receptor blockers (ARBs) combination therapies (*p* = 5.7 × 10^−4^), an association that is significantly stronger in females (*p*_*dif*__f_ = 0.01), and (2) with the use of non-steroid anti-inflammatory and antirheumatic products (*p* = 5.5 × 10^−4^). While these associations need to be confirmed in larger sample sizes, they suggest that these variants could play a role in diseases such as thrombocytopenia, hypertension, and chronic inflammation that are often observed in the more severe COVID-19 cases. Further investigation of these genetic variants in the context of COVID-19 is thus promising for better understanding of disease variability. Full results are available at https://covid19research.nl.

## Introduction

The recent outbreak of the coronavirus disease 2019 (COVID-19) caused by the SARS-CoV-2 virus has quickly become a pandemic and poses a great threat to public health. COVID-19 has a wide range of clinical manifestations: infected people can be asymptomatic, symptomatic with mild respiratory symptoms, or have severe pneumonia (Chen et al., 2020; Huang et al., 2020; Wu and McGoogan, 2020; Xu et al., 2020). Estimates based on reported cases from February 2020 in China indicated that ~20% of patients develop severe respiratory illness requiring hospitalization, and that overall mortality estimates are around 2.3% (Wu and McGoogan, 2020). These estimates are not fixed and are becoming more precise as more cases are reported, screened, and analyzed. Interestingly, there is high variability in these estimates when comparing countries and continents, as well as differences in COVID-19 severity between males and females and between different age groups (Chen et al., 2020; Wu and McGoogan, 2020; Zhou et al., 2020) [WHO Situation Report 70, from March 30, 2020]. Differences in response to SARS-CoV-2 infection between individuals and countries may be explained by diminished immune response in the elderly, comorbidities, or smoking habits (Guan et al., 2020), but severe COVID-19 cases have also been observed in young individuals, seemingly without risk factors. This indicates that most factors explaining COVID-19 severity are still unknown. It is therefore critical to understand the mechanisms behind COVID-19 severity in order to provide appropriate prevention measures and adequate triage strategies, guide the drug discovery process, and ultimately combat the SARS-CoV-2 pandemic.

The large variation in SARS-CoV-2 infection rates and COVID-19 severity could potentially be explained by genetic differences between hosts. While large-scale genetic studies of COVID-19 patients are being assembled, such as those coordinated by the COVID host genetics consortium (The COVID-19 Host Genetics Initiative, 2020; https://www.covid19hg.org/), it is worthwhile to evaluate the effects of genetic variants in genes involved in SARS-CoV-2 infection on human phenotypes, including quantitative traits, taking advantage of already existing cohorts. In fact, while quantitative phenotypes are not always directly associated with a disease, knowledge on the genetic variants that modulate these traits can improve our understanding of disease onset and the variability in symptoms. In one example of how this can work, genetic variants in the *BCL11A* gene were associated by genome-wide association studies (GWAS) to fetal hemoglobin (HbF) production in the general population (Menzel et al., 2007), and these genetic variants were subsequently found to modulate the severity of beta-thalassemia and sickle cell diseases (Lettre et al., 2008; Uda et al., 2008). This observation explained why certain individuals were naturally predisposed to mild symptoms of these diseases, while others had very severe clinical outcomes and benefitted from HbF increasing drugs. Therefore, understanding the role of genetic variants at genes essential for SARS-CoV-2 infection in human quantitative phenotypes is important to explain the observed variability in infection susceptibility and severity of COVID-19 and this understanding may suggest potential treatments.

Some factors that are necessary for SARS-CoV-2 infection are known (Hoffmann et al., 2020; Yan et al., 2020). Angiotensin converting enzyme 2 (*ACE2*) is necessary for the invasion of the virus into the host cell through viral spike proteins, and the transmembrane Serine Protease 2 (*TMPRSS2*) is necessary for the correct maturation of these same viral spike proteins that enter the cell through *ACE2* (Yan et al., 2020). According to the GWAS Catalog^1^, genetic variants in or near *TMPRSS2*, located on chromosome 21, are associated with susceptibility of prostate cancer and mortality rate in the population, while no associations have been reported for variants in or near *ACE2*. This can be partly explained by the fact that the *ACE2* gene is located on the X chromosome, a part of the genome that is often not analyzed by large scale genome wide association studies (GWAS) due to differences in analysis workflow with the autosomal chromosomes. Potential associations with human phenotypes near *ACE2* could have therefore been missed.

Here we investigated the association of genetic variants within or near (±100 Kb) *ACE2* and *TMPRSS2* transcripts through a phenome-wide association scan (PheWAS) in 36,339 volunteers from the Lifelines population cohort. We analyzed 72 quantitative phenotypes and the medication usage of 58 different drug categories in the entire cohort, and 92 protein levels in plasma, and 14 cytokines in a subset of ~600 individuals. The quantitative phenotypes selected are anthropometric traits and measurable parameters of lung, hearth, kidney, hematological, immune, and cardio-metabolic functions. Finally, in a sex-stratified anaysis we evaluated whether these variants were sex-specific or differed in their association between males and females to explore potential differences between sexes that could modulate SARS-CoV-2 infection.

## Materials and Methods

### Lifelines Cohort

The Lifelines cohort (Scholtens et al., 2015) is a multi-disciplinary prospective population-based cohort study, with a unique three generation design, that is examining the health and health-related behaviors of 167,729 individuals living in the North of the Netherlands. It was approved by the medical ethics committee of the University Medical Center Groningen and conducted in accordance with Helsinki Declaration Guidelines. All participants signed an informed consent form prior to enrollment. Lifelines employs a broad range of investigative procedures to assess the impact of biomedical, socio-demographic, behavioral, physical, and psychological factors on multi-morbidity, and complex genetics.

### Genotyping Data

A subset of 38,030 volunteers were genotyped using the Infinium Global Screening Array® (GSA) MultiEthnic Disease Version, according to manufacturer's instructions, at the Rotterdam genotyping center and the Department of Genetics, University Medical Center Groningen. We performed standard quality controls on both samples and markers, including removal of samples and variants with a low genotyping call rate (<99%), variants showing deviation from Hardy-Weinberg equilibrium (*p* < 1 × 10^−6^) or excess of Mendelian errors in families (>1% of the parent-offspring pairs), and samples with very high or low heterozygosity. We further checked and removed samples that did not show consistent information between reported sex and genotypes on the X chromosome, between reported familial information and observed identity-by-descent sharing with family members, and between genotypes available from this and previous studies (Francioli et al., 2015; Tigchelaar et al., 2015). A detailed description of the process can be found at the following link: https://covid19research.nl (van der Velde et al., 2019). After quality checks, a total of 36,339 samples and 571,420 autosomal and X-chromosome markers were available for analysis.

The genotyping dataset was then imputed using the Haplotype Reference Consortium (HRC) panel v1.1 at the Sanger imputation server^1^ (Consortium, 2015), and variants with an imputation quality score higher than 0.4 for variants with a minor allele frequency (MAF) > 0.01 and higher than 0.8 for rare variants (MAF < 0.01) were retained. 58.40% (21,241) of the 36,339 individuals whose genotype passed quality control were female, and the average age at phenotype collection was 39.9 years (±16.3 years).

### Phenotypes

Quantitative phenotypes were measured as previously described (Scholtens et al., 2015). We removed illegal zero or negative values for the “QRS,” “QT,” “HALB,” “MAP,” “MOP,” “EOP,” “BAP,” “U24HVOL,” “ALT,” “HR,” “EO,” “PQ,” “MO,” and “BA” phenotypes, and removed−999 values from the electrocardiogram phenotypes “P_AXIS,” “T_AXIS,” and “QRS_AXIS” (Table S1). Protein levels in plasma for 92 cardiovascular-related proteins were determined using Olink Proseek Multiplex CVD III panel (OLINK, Uppsala, Sweden), and concentrations of plasma citrulline and cytokines were measured by ProcartaPlex™ multiplex immunoassay (eBioscience, USA) as described previously (Zhernakova et al., 2016, 2018). Medication use was recorded based on drug packaging brought in by the participant's on their first visit to the Lifelines inclusion center. Registration of medication use in this way has been shown to be fairly to highly concordant with health record information (Sediq et al., 2018). After conversion to anatomical therapeutic chemical classification (ATC) codes, the first four letters (level 3) were used to define drug categories for association analyses. ATC codes with less than 100 observations were not considered for analysis, leaving 58 drug categories for analysis (Table S2).

### Statistical Analyses

We analyzed quantitative phenotypes using linear-mixed models implemented in SAIGEgds v1.0.0 so as to correct for familial relationships and cryptic population structure (Zheng et al., 2017; Zhou et al., 2018). For the X chromosome, genotypes in males were considered diploid. We tested the additive effect of 1,273 genetic variants within and near (±100 Kb) *ACE2* (chrX:15,579,156-15,620,271, GRCh37) and *TMPRSS2* (chr21:42,836,478-42,903,043, GRCh37) transcripts. These are all single-nucleotide polymorphisms (SNPs) with minor allele frequency (MAF) > 0.005 that were genotyped or imputed and that passed our quality controls as described above. Analysis through SAIGEgds was carried out for 72 quantitative phenotypes available for all, or a subset of the 36,339 samples (Table S1). Drug categories were analyzed as binary traits (1 = if medication currently in use, 0 otherwise) and restricted only to 1,240 genetic variants with MAF > 0.01. In both analyses age and sex were used as covariates. Inverse-normal transformation was applied to all quantitative traits prior to model fit. We searched for sex-specific effects by analyzing males and females separately (sex-stratified analyses), using only age as covariate and the same transformations as used for the analysis on the entire cohort. We also used the sex-stratified anaysis results to investigated differential genetic effects between sexes at suggestive associations identified in the combined analysis. This approach is typically used in small to moderate studies as an alternative to an analysis with an interaction term (Winkler et al., 2015).

**Table 1:** Most-significant associations with phenotypes at *ACE2* and *TMPRSS2* loci.

**Trait**	**Gene**	**rs.id**	**Chr:position**	**Ref/Alt**	**AF.Alt**	**Analysis**	***N***	**beta (*SE*)**	***p***
EO	*ACE2*	rs17264937	X:15647332	T/C	0.312	All	35,494	0.416 (0.11)	1.49 × 10^−4^
						Males only	14,751	0.357 (0.146)	0.0146
						Females only	20,743	0.531 (0.159)	8.17 × 10^−4^
TGL	*ACE2*	rs5980163	X:15521666	C/G	0.016	All	36,112	0.071 (0.019)	1.63 × 10^−4^
						Males only	15,004	0.108 (0.031)	4.12 × 10^−4^
						Females only	21,108	0.014 (0.021)	0.488
CHIT1	*TMPRSS2*	rs150965978	21:42942652	C/A	0.063	All	526	−0.630 (0.131)	2.13 × 10^−6^
						Males only	241	−0.502 (0.209)	0.017
						Females only	285	−0.555 (0.175)	0.002
TR	*TMPRSS2*	rs28401567	21:42951813	C/T	0.166	All	36,049	−2.50 (0.583)	1.77 × 10^−5^
						Males only	14,975	−1.82 (0.788)	0.0210
						Females only	21,074	−2.74 (0.775)	4.04 × 10^−4^

*The table reports summary statistics for the two most-associated phenotypes at ACE2 and TMPRSS2 loci. Positions refer to genome build GRCh 37. Beta indicates the effect for each copy of the alternative allele, in standard deviation units. Ref, reference allele; Alt, alternative allele; AF.Alt, alternative allele frequency; SE, Standard Error; EO, Eosinophils; TGL, triglycerides; CHIT1, plasma levels of CHIT1 protein; TR, thrombocytes*.

The 92 circulating plasma proteins and 14 cytokines were measured in a small subset of unrelated individuals and thus did not require correction for familial relationships. These were analyzed using PLINK v2.00a3LM. We performed the association mapping with both sexes jointly, or separately as described above, and using inverse-normal transformation on the traits. We analyzed each variant and trait combination with or without the inclusion of age and sex covariates, as some genetic variants were too highly correlated due to the small sample size, and thus an estimate with covariates included in the model was not possible. To evaluate the statistical power of our study we used the package GeneticsDesign in R (Weilang et al., 2019). For quantitative variable analyses, we used the function *GeneticPower.Quantitative.Numeric()* and calculated the minimum detectable additive effect (variance explained) with 80% power and at a significance threshold of 5 × 10^−8^, for an increasing number of samples up to 36,339 (our study size). For binary variables analysis, we used the function *GPC.default()* and calculated the minimum detectable additive effect size (genotype relative risk) with 80% power and at a significance threshold of 5 × 10^−8^, for an increasing number of cases in a cohort of 36,339 and for a risk allele frequency varying from 0.05 to 0.5. We set the number of cases up to 4,000 to reflect the maximum number of users for the analyzed drug categories in our study. We also assumed that the causal variant was included in our genotyping data set, therefore we constrained full linkage disequilibrium (Dprime = 1) with the tag marker. Since disease prevalence (*pD*) could also impact power, we calculated the minimum detectable effect for *pD* varying from 1 to 20%.

## Results

### Quantitative Phenotypes

**Figure 1:**
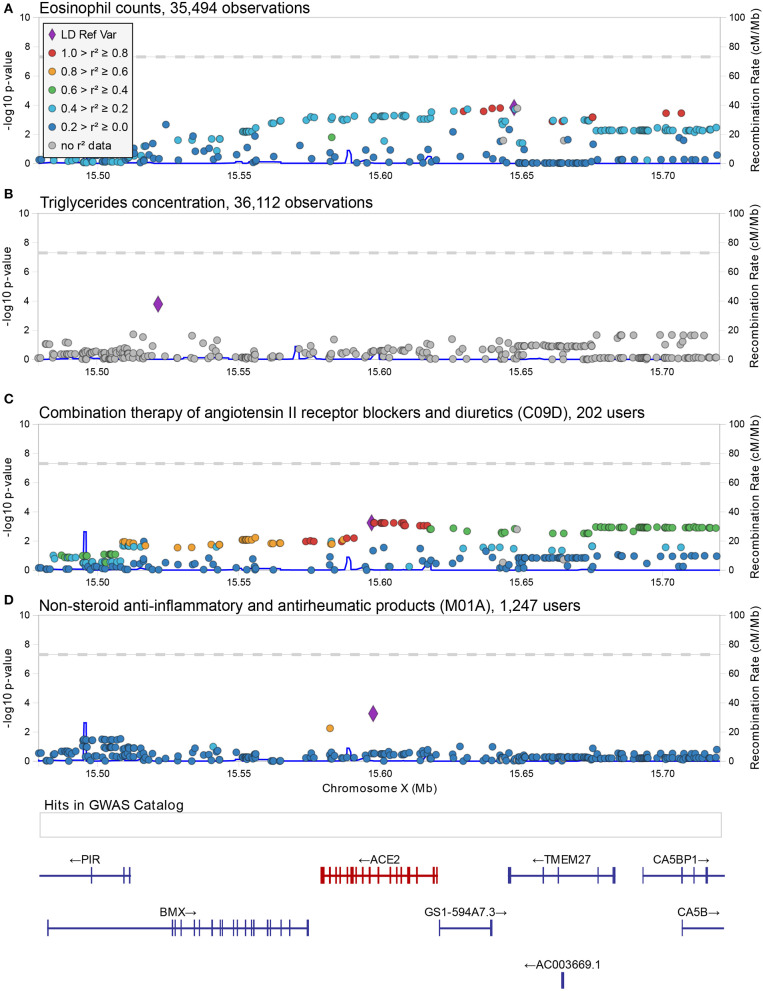
Regional associations plot at the *ACE2* locus. Graphical representation of the association results at the *ACE2* locus for the SNP-trait associations reported in Table 1 **(A,B)** and Table 2 **(C,D)**. In each panel, each dot represents a genetic variant, and shown is the association strength (expressed as negative log10 *P*-values, *Y*-axis) vs. the genomic position (on the hg19/GRCh37 genomic build, *X*-axis). The strongest associated variant is depicted with a purple diamond, while other variants are color-coded to reflect their linkage disequilibrium with it (taken from pairwise *r*^2^ values calculated from the 1,000 Genomes Europeans). A legend for color-coding is provided in **(A)**. In **(D)**, an additional box shows the location of associations reported in the GWAS catalog (no associations were reported at this locus) and below this box the location of genes is shown with specification of exons and direction of transcription. This figure was drawn using LocusZoom web tool (Pruim et al., 2010).

**Figure 2:**
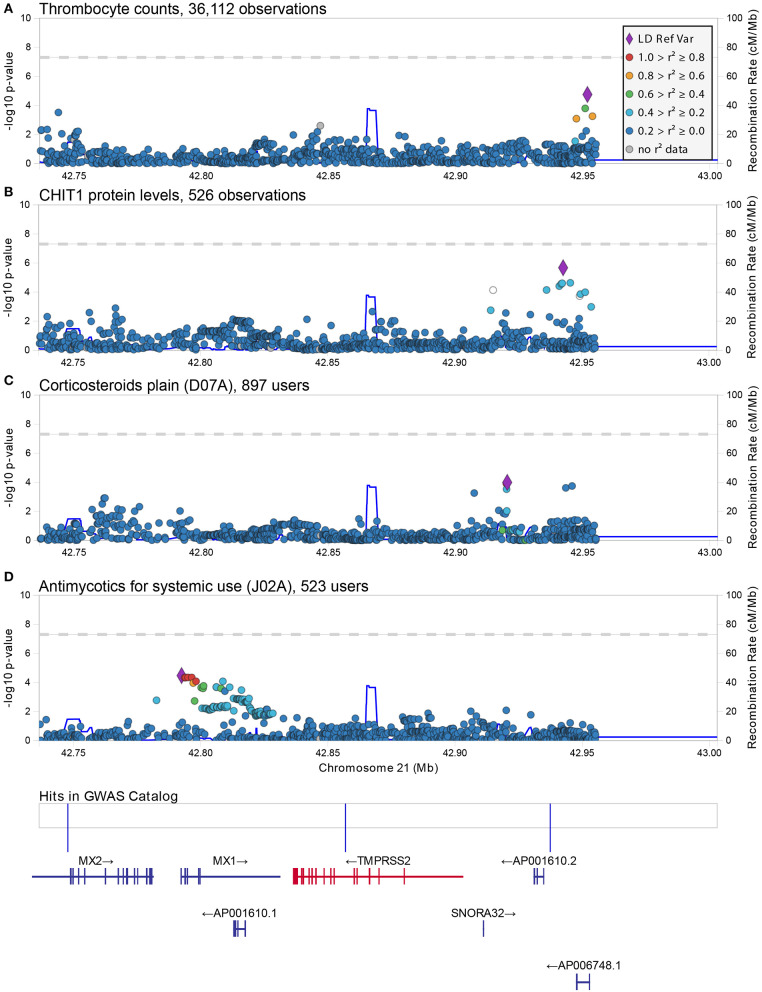
Regional associations plot at the *TMPRSS2* locus. Graphical representation of the association results at the *TMPRSS2* locus for the SNP-trait associations reported in Table 1 **(A,B)** and Table 2 **(C,D)**. In each panel, each dot represents a genetic variant and shown is the association strength (expressed as negative log10 *P*-values, *Y*-axis) vs. the genomic position (on the hg19/GRCh37 genomic build, *X*-axis). The strongest associated variant is depicted with a purple diamond, while other variants are color-coded to reflect their linkage disequilibrium with it (taken from pairwise *r*^2^ values calculated from the 1,000 Genomes Europeans). A legend for color-coding is provided in **(A)**. In **(D)**, an additional box shows the location of associations reported in the GWAS catalog (associations here reported from left to right are: melanoma, age-related diseases and mortality, and prostate cancer) and below this box the location of genes is shown with specification of exons and direction of transcription This figure was drawn using LocusZoom web tool (Pruim et al., 2010).

Using a linear-mixed model, we analyzed 1,273 common and low frequency (MAF > 0.005) genetic variants in and near (+/−100Kb) *ACE2* and *TMPRSS2* transcripts for association with 178 quantitative traits (Table S1). None were found to be significant at the standard genome-wide level (*p* = 5 × 10^−8^) or at the Benjamin-Hochberg false discovery rate (FDR < 0.1). The most significant associations found with quantitative traits at the *ACE2* locus were with triglycerides (rs5980163, *p* = 1.6 × 10^−4^) and with the eosinophil counts (rs17264937, *p* = 1.5 × 10^−4^) (Table 1) (Figure 1). The strongest associations at the *TMPRSS2* locus were with plasma levels of CHIT1 (rs150965978, *p* = 2.1 × 10^−6^) and thrombocytes (rs28401567, *p* = 1.7 × 10^−5^) (Table 1) (Figure 2). Only the association at rs5980163 with triglycerides at *ACE2* showed a differential effect between males and females (Cochran *Q*-test *p*_*diff*_ = 0.01), with most of the signal being attributable to males, although the association remains only suggestive (*p* = 4.12 × 10^−4^). We did not find any signal that was restricted to either males or females (*p* > 1 × 10^−6^ for all associations in the sex-stratified analyses). The SNP-trait associations reported in Table 1 were not replicated in the UK Biobank, based on summary statistics from an analysis that included at least 343,992 samples^1^ (all *p* > 0.05). No replication was observed also for the association with CHIT1 plasma levels using results from the INTERVAL study (Sun et al., 2018).

### Medication Use

For this analysis, we focused on 1,240 variants with MAF > 0.01. As with the quantitative phenotypes, none of the genetic variants showed genome-wide significant association with medication use (Table S2). The strongest associations at the *ACE2* locus were observed for the group of drugs that contains non-steroid anti-inflammatory and antirheumatic products (NSAIDs) (ATC = M01A) [odds ratio (OR) = 1.34, 95% C.I. = 1.14–1.58, *p* = 5.5 × 10^−4^ for the G allele of rs4646190] (Table 2) (Figure 1), and for the group that contains angiotensin II receptor blockers (ARBs) in combination with other antihypertensive drugs (ATC = C09D) (OR = 1.35, 95% C.I. = 1.14–1.62 *p* = 5.7 × 10^−4^ for the T allele of rs4646156) (Table 2) (Figure 1). These SNPs are both located in intron eight of the *ACE2* transcript and only 525 bp apart, but they are not in linkage disequilibrium (*r*^2^ = 0.05 in 1,000 Genomes Europeans).

**Table 2:** Most-significant associations with medications use at *ACE2* and *TMPRSS2* loci.

**Drug ATC code**	**Gene**	**rs.id**	**Chr:position**	**Ref/Alt**	**AF.Alt**	**Analysis**	***N* users**	**OR (95%C.I.)**	***p***
M01A	*ACE2*	rs4646190	X:15597568	A/G	0.045	All	1,274	1.34 (1.14–1.58)	5.5 × 10^−4^
						Males only	412	1.54 (1.22–1.97)	3.7 × 10^−4^
						Females only	862	1.16 (0.92–1.46)	0.213
C09D	*ACE2*	rs4646156	X:15597043	A/T	0.646	All	202	1.36 (1.14–1.62)	5.71 × 10^−4^
						Males only	88	1.14 (0.92–1.42)	0.231
						Females only	114	1.78 (1.35–2.34)	4.69 × 10^−5^
J02A	*TMPRSS2*	rs457274	21:42792485	C/G	0.406	All	523	1.33 (1.16–1.51)	3.36 × 10^−4^
						Males only	234	1.23 (1.01–1.50)	0.041
						Females only	289	1.41 (1.25–1.60)	1.46 × 10^−4^
D07A	*TMPRSS2*	rs9975623	21:42920296	A/G	0.304	All	897	1.23 (1.07–1.36)	1.04 × 10^−4^
						Males only	348	1.29 (1.09–1.51)	2.52 × 10^−3^
						Females only	549	1.19 (1.04–1.35)	0.011

*The table reports summary statistics for the two most-associated drug categories (given in ATC codes) at ACE2 and TMPRSS2 loci. Positions refer to genome build GRCh 37. The Odds Ratio refers to the alternative allele. Ref, reference allele; Alt, alternative allele; AF.alt, alternative allele frequency; OR, Odds ratio; M01A, Anti-inflammatory and antirheumatic products, non-steroids; C09D, Angiotensin II receptor blockers (ARBs), combinations; J02A, Antimycotics for systemic use; D07A, Corticosteroids, plain*.

NSAIDs are used for treating pain, fever and inflammation, and include ibuprofen. The significance of rs4646190 was stronger in males (*p* = 3.7 × 10^−4^) than in females (*p* = 0.08), but the effect sizes were not statistically different (*p*_*diff*_ = 0.054).

The second group of drugs encodes for a combined therapy used to treat hypertension. Combination therapy of ARBs with other hypertensive drugs is usually initiated as a second option when the antihypertensive effect of an ARB alone is not sufficient (Ram, 2004; Flack, 2007). Our results indicate that individuals carrying at least one T allele at the rs4646156 polymorphism were more likely to take this combined therapy compared to individuals with the other allele. The effect of this SNP was also not significant when considering only ARBs intake (ATC = C09C, *p* = 0.66). Thus, the association with ARB combination therapies could indicate that individuals in whom it is difficult to manage hypertension may be genetically predisposed to this state by rs4646156. Interestingly, when analyzing males and females separately, we found that the signal of rs4646156 on ARB combination therapy was mostly attributable to females, even when accounting for differences in number of users (OR = 1.78, 95% C.I. = 1.35–2.34, *p* = 4.7 × 10^−5^ in females vs. OR = 1.14, 95% C.I. = 0.92–1.42, *p* = 0.23 in males, *p*_*diff*_ = 0.01).

We reiterate that none of these associations (in the combined and in the sex-specific analyses) meet either the genome-wide or FDR thresholds for significance. To confirm these findings larger sample sizes are necessary.

The strongest associations at the *TMPRSS2* locus were observed for the group of drugs containing antimycotics prescriptions (ATC = J02A) (*p* = 3.65 × 10^−5^) and for corticosteroids (ATC = D07A) (*p* = 1.0 × 10^−4^) (Table 2) (Figure 2). No significant difference in effect size between sexes was observed for these two associations (*p*_*diff*_ > 0.2). These SNPs were independent from each other and from the top associations with quantitative traits described in Table 1.

We attempted to validate our findings on medication use using again the UK Biobank public GWAS summary statistics^1^, although their data refers to the use of individual medications rather than drug categories. When considering the medications most commonly used (>1,000 users in the UK Biobank cohort) in the categories of interest (M01A, C09D, J02A, and D07A), we found nominal association with the same direction of effects only for glucosamine use (ATC = M01A, *p* = 0.002 in the combined analysis and *p* = 0.008 in males only) and with candesartan cilexetin (ATC = C09D, *p* = 0.008 in females only) (Table S3). A similar detailed analysis in our cohort was underpowered to detect an association signal for single medications of C09D and M01A categories (Table S4). This lack of replication could be attributable to differences in medication usage reporting between studies. While both are based on self-reported information, in the Lifelines study records are confirmed by medication packaging collected by a nurse during the recruitment.

## Discussion

Recent studies have demonstrated that SARS-CoV-2 uses ACE2 as the key receptor to invade cells (Yan et al., 2020) and that ACE2-mediated cell invasion is enhanced by *TMPRSS2* expression (Hoffmann et al., 2020). Genetic variations in these two genes that interfere with the gene function may thus be involved in the observed variability of SARS-CoV-2 susceptibility and COVID-19 severity. The association of these genetic variants with human phenotypes in the general population may suggest potential treatments and help to better identify at-risk individuals. Here we used a cohort of 36,339 individuals from the Lifelines general population cohort to investigate the impact of variants near and within these two genes on 178 quantitative traits including measurable parameters of lung, hearth, kidney, hematological, immune, and cardio-metabolic functions.

We found no significant evidence that common and low frequency variants in these loci were associated with the measured quantitative traits in the general population. We did observe suggestive signals for phenotypes (triglycerides and thrombocytes) that are involved in cardiovascular diseases, which are considered risk factors for COVID-19 diseases (Wu and McGoogan, 2020), but none of the genetic variants reached statistical significance despite our large sample size. Nevertheless, we cannot exclude a role of these variants in the regulation of COVID-19 severity through other relevant phenotypes such as specific immune cell types or cytokine levels that were not measured in our cohort.

To evaluate the effect of genetic variation in clinically relevant phenotypes, we investigated the association of genetics with medication use. We observed a marginal association of variants within *ACE2* with use of ARBs combination therapy (ATC = C09D, rs4646156) and with use of non-steroidal anti-inflammatory and antirheumatic drugs (NSAIDs, rs4646190). Interestingly, a marginal association with ARBs (C09C category) was also observed at the *TMPRSS2* locus (rs75833467, *p* = 3.5 × 10^−4^). These results are intriguing considering the current debate about whether the use of ARBs and NSAIDs could worsen COVID-19 severity (Kuster et al., 2020; Little, 2020; Russell et al., 2020), and their potential effect on increasing *ACE2* expression. No significant associations were found for these variants with blood pressure measurements or inflammatory markers in our cohort (*p* < 0.05), not even when the use of such drugs were added as covariates (data not shown). Association with diastolic and systolic blood pressure was also not observed in the large UK Biobank cohort. Thus, these variants are likely to be associated only with clinical conditions such as hypertension and chronic inflammation or with a better drug response. It has to be noted that our sample size allowed sufficient statistical power to detect genetic variants with small effects (down to 0.001 of variance explained), and thus we are confident in claiming lack of association at *TMPRS22* and *ACE2* loci with the quantitative phenotypes assessed. For analyses on medication usage we were instead sufficiently powered to find small effects (genotype relative risk ~1.1) only for very common SNPs (frequency >0.3), but we are underpowered for smaller effects and, in general, at less common variants (Figure S1). Therefore, our suggestive results for medication usage could indicate a real effect for which we were underpowered to find a genome-wide significance evidence. Further exploration of these associations is needed.

ARBs are the preferred alternative for patients who experience ACE-inhibitor induced coughing. However, as rs4646156 is not associated with this adverse drug reaction (ADR), our results are likely independent of the switch to ARBs due to ACE-inhibitor induced coughing (Mas et al., 2011). Interestingly, the association of this SNP with ARBs was specific to ARBs combination therapy, thus pointing to individuals with difficult-to-manage hypertension. The major allele (T) of rs4646156 has different frequencies across populations: 0.653 in Europeans, 0.997 in East Asians and 0.797 South Asians, according to 1,000 Genomes^1^. Likewise, the G allele at the rs4646190 SNP, associated with a higher probability of NSAIDs use, shows substantial different frequencies among populations. It is mostly absent in Asians but not in Europeans: 0.03 in Europeans, 0 in East Asians and 0.003 in South Asians, according to 1,000 Genomes^1^.

The suggestive genetics associations we find for NSAIDs and ARBs combination therapy indicate that, depending on their genotype, certain individuals are predisposed to take these drugs, and thus to suffer from hypertension and chronic inflammation, diseases often described among COVID-19 comorbidities. This, together with the observed different allele frequencies across continents and the sex-related differential effects could explain the observed variation in COVID-19 severity between countries and sexes. Unfortunately, we could only speculate around this hypothesis as this study is not suited to prove that these genetic associations are directly related to SARS-CoV-2 susceptibility or COVID-19 severity, nor we can determine if ARBs or NSAIDs improve or worsen COVID-19 severity. A role of ARBs in worsing severity seems however unlikely (Gill et al., 2020; Mancia et al., 2020; Mehra et al., 2020).

We acknowledge the following limitations in our study. First, only age and sex were used as covariates in our analyses, which may not be sufficient to correct for confounders for all traits, such as drug usage or diseases, although the effect of these confounders should be mitigated by our sample size. Secondly, our analyses on medication use are underpowered given the limited number of individuals in the general population who use the medications that we tested, and thus none of the associations found here met the multiple-testing adjusted significance. Third, our results for medication use did not include low frequency and none of the analysis include rare variants (MAF < 0.005) which could still be relevant. Fourth, while we can speculate about potential connections of our results with current knowledge of COVID-19, longitudinal and well-characterized data on patients is needed to further explore our hypothesis.

In conclusion we carried out an extensive screening of potential genetic associations at common and low frequency variants in the *ACE2* and *TMPRSS2* genes, and found a lack of substantial effect in human quantitative phenotype variation in the general population. Genetic analyses in more phenotypes are needed to evaluate their functional role in other physiological processes.

Finally, since genetic variation in other genes, for example those involved in regulating the immune system, could also be important in determining SARS-CoV-2 susceptibility and disease severity, large scale genetic initiatives like the COVID-19 host genetics consortium (The COVID-19 Host Genetics Initiative, 2020; https://www.covid19hg.com/) that directly involve patients with COVID-19 and deeply characterization of genomes and phenotypes are urgently needed.

## Data Availability Statement

The data analyzed in this study was obtained from the Lifelines biobank, under project application number OV18_0463. Requests to access this dataset should be directed to Lifelines Research Office (research@lifelines.nl). Full summary statistics of the results are available at https://covid19research.nl.

## Ethics Statement

The studies involving human participants were reviewed and approved by the medical ethics committee of the University Medical Center Groningen and conducted in accordance with Helsinki Declaration Guidelines. Written informed consent to participate in this study was provided by the participants' legal guardian/next of kin.

## Author Contributions

EL and AG performed statistical analyses. EL, AG, PL, PD, AZ, and SS interpreted results. MG and MS provided computing infrastructure and web portal. Lifelines Cohort Study, LF, CW, JF, and AZ provided access to the data. EL, AG, and SS wrote the manuscript draft with critical input from PL, LF, CW, PD, JF, and AZ. All authors read and approved the manuscript.

## Lifelines Cohort Study—Group Authors Genetics

Raul Aguirre-Gamboa (1), PD (1), LF (1), Jan A Kuivenhoven (2), EL (1), Ilja M Nolte (3), SS (1), Harold Snieder (3), MS (1), Judith M Vonk (3), CW(1)

(1) Department of Genetics, University of Groningen, University Medical Center Groningen, Netherlands.
(2) Department of Pediatrics, University of Groningen, University Medical Center Groningen, Netherlands.
(3)Department of Epidemiology, University of Groningen, University Medical Center Groningen, Netherlands.

## Conflict of Interest

The authors declare that the research was conducted in the absence of any commercial or financial relationships that could be construed as a potential conflict of interest. The reviewer JK declared a shared affiliation with no collaboration with one of the authors PD to the handling editor at time of review.

## Footnotes

^1^1000 Genomes study: https://www.internationalgenome.org/; Sanger imputation server: https://imputation.sanger.ac.uk; GWAS catalogue: https://www.ebi.ac.uk/gwas/home (accessed on April 6, 2020); UK biobank all phenotype associations: http://www.nealelab.is/uk-biobank.

## Supplementary Material

Figure S1 Statistical power estimates. **(A–D)** show statistical power estimates for binary trait analyses, and **(E)** for quantitative trait analyses. In **(A–D)**, each line shows the minimum detectable additive effect size (genotype relative risk, *Y*-axis) with 80% power and at a significance threshold of 5 × 10^−8^, for an increasing number of cases in a cohort of 36,339 (*X*-axis). The different lines depict these values when risk allele frequency varies from 0.05 to 0.5. The label of the *X*-axis indicates the disease prevalence (*pD*) considered in each panel. In **(E)**, the line shows the minimum detectable additive effect (variance explained, *Y*-axis) with 80% power and at a significance threshold of 5 × 10^−8^, for an increasing number of samples up to 36,339 (*X*-axis).
