# Supplementary figures and images for: Lack of Association Between Genetic Variants at ACE2 and TMPRSS2 Genes Involved in SARS-CoV-2 Infection and Human Quantitative Phenotypes
Source: Front Genet. 2020 Jun 8;11:613. doi: 10.3389/fgene.2020.00613 (PMC7295011; doi:10.3389/fgene.2020.00613)

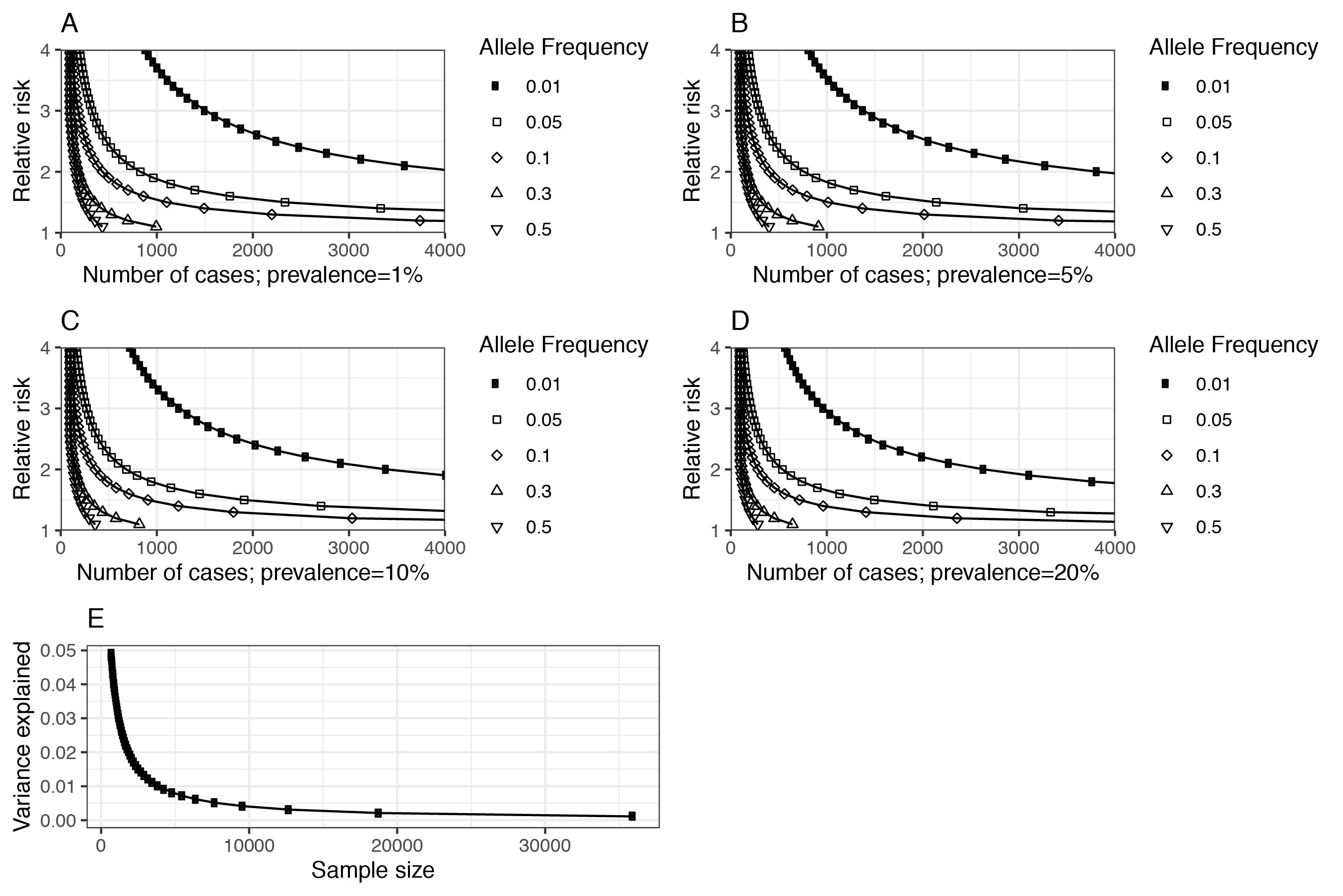

Supplement: Figure S1 — Statistical power estimates. (A–D) show statistical power estimates for binary trait analyses, and (E) for quantitative trait analyses. In (A–D), each line shows the minimum detectable additive effect size (genotype relative risk, Y-axis) with 80% power and at a significance threshold of 5 × 10^−8^, for an increasing number of cases in a cohort of 36,339 (X-axis). The different lines depict these values when risk allele frequency varies from 0.05 to 0.5. The label of the X-axis indicates the disease prevalence (pD) considered in each panel. In (E), the line shows the minimum detectable additive effect (variance explained, Y-axis) with 80% power and at a significance threshold of 5 × 10^−8^, for an increasing number of samples up to 36,339 (X-axis). [file Image_1.jpg]
